# Effect of Renal Denervation for the Management of Heart Rate in Patients With Hypertension: A Systematic Review and Meta-Analysis

**DOI:** 10.3389/fcvm.2021.810321

**Published:** 2022-01-17

**Authors:** Le Li, Yulong Xiong, Zhao Hu, Yan Yao

**Affiliations:** Fu Wai Hospital, National Center for Cardiovascular Diseases, Peking Union Medical College, Chinese Academy of Medical Sciences, Beijing, China

**Keywords:** renal denervation, hypertension, heart rate, meta-analysis, sympathetic nerve

## Abstract

**Objective:**

The effect of renal denervation (RDN) on heart rate (HR) in patients with hypertension had been investigated in many studies, but the results were inconsistent. This meta-analysis was performed to evaluate the efficacy of RDN on HR control.

**Methods:**

Databases, such as PubMed, EMBASE, Cochrane, and ClinicalTrials.gov, were searched until September 2021. Randomized controlled trials (RCTs) or non-RCTs of RDN in hypertensive patients with outcome indicators, such as HR, were selected. Weighted mean difference (WMD) was calculated for evaluating the changes in HR from baseline using fixed-effects or random-effects models. The Spearman's correlation coefficients were used to identify the relationship between the changes of HR and systolic blood pressure (SBP).

**Results:**

In the current meta-analysis, 681 subjects from 16 individual studies were included. This study showed that RDN could reduce office HR in patients with hypertension [WMD = −1.93 (95% *CI*: −3.00 to −0.85, *p* < 0.001)]. In addition, 24-h HR and daytime HR were decreased after RDN [WMD = −1.73 (95% *CI*: −3.51 to −0.31, *p* = 0.017) and −2.67 (95% *CI*: −5.02 to −0.32, *p* = 0.026) respectively], but nighttime HR was not significantly influenced by RDN (WMD = −2.08, 95% *CI*: −4.57 to 0.42, *p* = 0.103). We found that the reduction of HR was highly related to the decrease of SBP (*r* = 0.658, *p* < 0.05).

**Conclusion:**

Renal denervation could reduce office, 24-h, and daytime HR, but does not affect nighttime HR. And the effect is highly associated with blood pressure (BP) control.

**Systematic Review Registration:**

https://www.crd.york.ac.uk/PROSPERO, identifier: CRD42021283065.

## Introduction

The prevalence of hypertension is increasing globally due to the high proportion of elder and obese people (1). Although relevant drugs are constantly updated and optimized for optimal treatment strategies for hypertension, the rate of hypertension control is still unsatisfied due to issues of drug efficacy, safety, and compliance, with ~10% of patients suffered from resistant hypertension (2). Device-based interventions present a new treatment option for patients with refractory hypertension (3). Renal denervation (RDN) could reduce blood pressure (BP) by destructing sympathetic nerve fibers and then suppressing the sympathetic overexcitation, and the effectiveness and safety have been confirmed in several clinical studies (4–6).

Heart rate (HR) is regulated by both sympathetic and parasympathetic nerves, with sympathetic overactivation causing an increase in HR and parasympathetic nerves acting in the opposite direction (7). Increased resting HR is associated with high cardiovascular morbidity and mortality (8). In a high-risk population where 75% had hypertension, increased HR is an independent risk factor for the development of heart failure (9). RDN can reduce BP by suppressing overexcited sympathetic activity through transcatheter renal artery ablation, which theoretically has a role in HR control. Böhm et al. (10) suggested that the reduction of 24-h HR was more significant in patients treated with RDN than in the group received sham procedure (−2.5 vs. −0.3 bpm, *p* = 0.003) in patients with resistant hypertension. However, a previous study has found that RDN may not have an effect on HR (11). The role of RDN in reducing HR in patients with hypertension is still controversial. In this study, a meta-analysis of RDN was conducted in aiming to identify the effect on HR control in hypertensive patients.

## Methods

### Literature Search Strategy

The primary search was conducted using the terms “renal denervation,” “renal sympathetic denervation,” “denervation,” “hypertension,” and “high blood pressure” as the subject words, combined with their entry terms and abbreviated terms to search the electronic databases of PubMed, EMBASE, Cochrane, and ClinicalTrials.gov. To avoid the miss of relevant articles, we searched the conference papers, degree papers, and other gray literature. The search end date was September 2021. The search language was only English. Manual retrieval included the references of published literature.

### Inclusion and Exclusion Criteria

Inclusion criteria: (1) randomized controlled trial (RCT) or non-RCT intervention trial; (2) the participants were adults aged 18 years or older with the history of hypertension; (3) the intervention strategy was RDN; and (4) the change of HR was reported in the outcomes.

Exclusion criteria: articles were excluded for the following: (1) reviews, case reports, and comments; (2) duplicate publications; and (3) incomplete or missing study data.

### Literature Screening and Quality Assessment

Literature screening was performed by two investigators (ZH and YLX) according to the inclusion and exclusion criteria, and when there was disagreement, a third investigator (LL) participated in the discussion and reached an agreement. The Cochrane Collaboration's tool for assessing risk of bias was used to evaluate the quality of the RCT literature. In addition, the quality of non-RCTs was assessed by the methodological index for non-randomized studies (MINORS) tool. Details of the Cochrane Collaboration's tool (12) and MINORS (13) have been described elsewhere.

### Data Extraction

Data from the eligible articles were extracted by three investigators (LL, ZH, and YLX) using a standard protocol. Two researchers (ZH and YLX) were responsible for collecting data, and the third researcher (LL) served as an arbitrator to resolve any divergence between them. The first author, published year, country, follow-up period, types of RDN device of the literature, and the age, gender, body mass index (BMI), history of coronary artery disease (CAD), current use of beta-blocker, baseline, and change of HR, and office systolic blood pressure (OSBP) of participants were collected in this meta-analysis. The primary outcome was the change of office HR, and the second outcomes were the change of 24 h-HR, daytime HR, and nighttime HR. The change of OSBP was also collected in this meta-analysis to verify the correlation between the change of HR and BP.

### Statistical Analyses

The meta-analysis was performed using RevMan 5.3 and Stata 15.0 (Stata Corp, College Station, TX, USA), and the change of HR and BP was shown as weighted mean difference (WMD) ± SD, and differences were considered statistically significant at *p* < 0.05. Heterogeneity of included studies was assessed by the *I*^2^ test, with *I*^2^ < 30% being low heterogeneity, 30–50% being moderate heterogeneity, and >50% being high heterogeneity. A fixed-effects model was used if there was no statistical heterogeneity among the results of studies, and a random-effects model was used if there was high heterogeneity. When high heterogeneity existed, methods, such as meta-regression analysis and subgroup analysis, were used. The sensitivity analysis was realized by excluding a single study in turn and then analyzing the remaining studies. The Begg's funnel plot and Egger's test were used to evaluate the publication bias of the literature. Meta-analysis results were presented with forest plots.

The relevant data which were not presented in the article were sought from authors. The WMD in each group can be obtained by subtracting the post-intervention mean from the baseline mean if it has not been presented explicitly (14). Additionally, the alternative technique was used for calculating the missing change SD when only baseline SD and final SD were available (15). If the literature reported the *CI* of WMD instead of SD, we used a function to calculate SD (14).

## Results

### Literature Search Results

**Figure 1:**
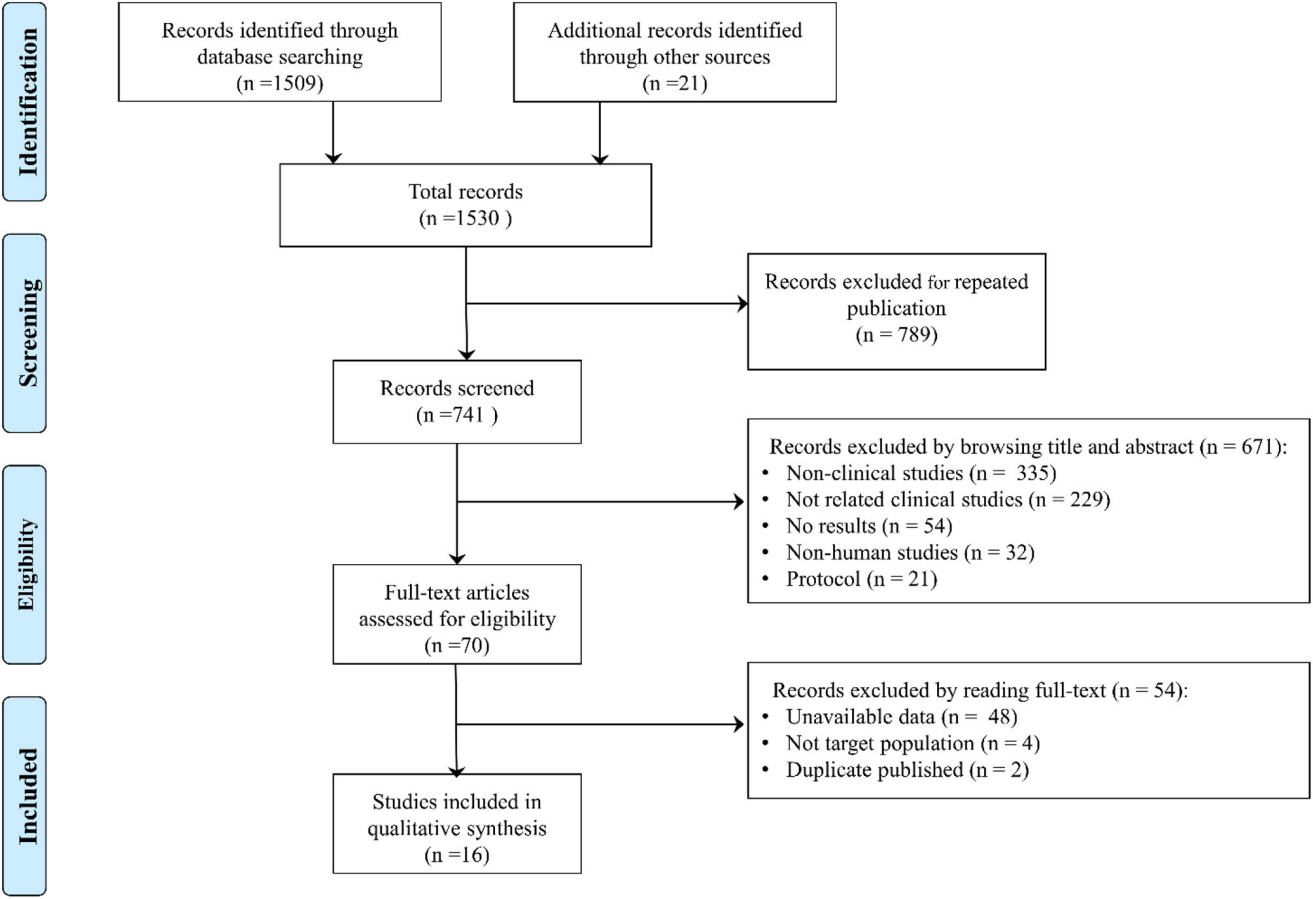
PRISMA flow diagram of the literature search process.

In total, 1,530 studies were retrieved, such as 1,509 studies retrieved by database and 21 studies retrieved by manual retrieval, and 741 studies were obtained after removing duplicate publications. By reading the title and abstract, 671 articles were excluded (335 were non-clinical studies, such as reviews, meta-analysis, and comments; 229 were not related articles; 54 did not report results; 32 were non-human studies; and 21 were protocols) according to the inclusion and exclusion criteria. A total of 70 articles were assessed by browsing full-texts, and 54 records were excluded (48 did not report the related data; 4 were not the target study population; and 2 were duplicate articles). Finally, 16 eligible clinical trials were included in the meta-analysis (10, 11, 16–29) (Figure 1).

### Included Literature Characteristics and Results

The included articles were published in recent years (2011–2020). The most heart rhythm of the participants was sinus rhythm, except for the Kiuchi et al. (20), in which the population suffered from paroxysmal atrial fibrillation. The device for RDN included a radiofrequency catheter (Symplicity Flex, Ardian Inc, Palo Alto, CA; Spyral, Medtronic Inc, Galway, Ireland; Therapy Cool Path, St. Jude Medical Inc, MN, USA) and an endovascular ultrasound catheter (Kona Surround Sound, Kona Medical Inc, Washington DC, USA; Paradise, ReCor Medical Inc, Palo Alto, CA, USA). The approach of RDN was ablation of the bilateral main renal artery, and in the literature of Böhm et al. (10), main renal artery plus side branches ablation was used for RDN procedure. All included trials were followed-up for at least 3 months. The characteristics of the included studies were summarized in Table 1. Table 2 showed the interventions and the RDN procedure details of treatment and control groups.

**Table 1:** Baseline characteristic of included studies.

**References**	**Year**	**Region**	**Sample size**	**Age**	**Follow up**	**Male**	**BMI**	**CAD**	**Beta-blocker**	**Study type**
Mahfound et al. (16)	2011	Germany	37	58.7	3M	79%	31.3	NA	89%	RCT
Ukena et al. (17)	2011	Germany	37	59.1	3M	68%	31.8	11%	89%	RCT
Ukena et al. (18)	2013	Germany	127	62.2	3M	58%	31.4	10%	88%	Non-RCT
Fengler et al. (19)	2016	Germany	22	57	6M	73%	NA	64%	91%	RCT
Kiuchi et al. (20)	2016	Brazil	39	60	6M	62%	24.9	NA	56%	Non-RCT
Courand et al. (21)	2017	France	52	54.5	6M	61.9%	30.8	NA	NA	RCT
Peters et al. (11)	2017	Denmark	26	54	6M	65%	28	4%	92%	RCT
Rosa et al. (22)	2017	Czech	52	56	24M	77%	31.2	6%	NA	RCT
Celinska et al. (23)	2018	Poland	30	55.9	3M	80%	34.7	40%	93%	RCT
Engholm et al. (24)	2018	Denmark	29	54.1	6M	72%	27.9	7%	83%	RCT
Schmieder et al. (25)	2018	Germany	42	60.3	3M	81%	29.9	5%	64%	RCT
Sexena et al. (26)	2018	Britain	12	57.2	6M	75%	31.7	33%	33%	RCT
Oliveras et al. (27)	2018	Spain	11	61.9	6M	55%	33.7	18%	55%	RCT
Böhm et al. (10)	2019	Germany	35	55.8	3M	68%	29.8	0%	0%	RCT
Lurz et al. (28)	2020	Germany	25	64.4	6M	NA	NA	64%	NA	RCT
Ukena et al. (29)	2020	Germany	105	63.5	6M	67%	NA	27%	83%	Non-RCT

*BMI, body mass index; CAD, coronary artery disease; M, month; NA, not available; RCT, randomized controlled trial*.

**Table 2:** Comparisons of intervention between included studies.

**References**	**Year**	**Treatment group**	**Control group**	**RDN procedure**				
				**Device**	**Ablation point**	**Time per point**	**Ablation power**	**Ablation rounds per artery**
Mahfound et al. (16)	2011	Bilateral RDN plus baseline AHM	Baseline AHM	Symplicity Flex	MRA	≤2 mins	≤8 W	4–6
Ukena et al. (17)	2011	Bilateral RDN plus baseline AHM	Baseline AHM	Symplicity Flex	MRA	≤2 mins	≤8 W	4–6
Ukena et al. (18)	2013	Bilateral RDN plus baseline AHM	NA	Symplicity Flex	MRA	≤2 mins	≤8 W	4–6
Fengler et al. (19)	2016	Bilateral RDN plus baseline AHM	Sham procedure plus baseline AHM	Symplicity Flex	MRA	≤2 mins	NA	4–6
Kiuchi et al. (20)	2016	Bilateral RDN plus PVI plus baseline AHM	PVI plus baseline AHM	Therapy Cool Path	MRA	1 min	10 W	≥ 4
Courand et al. (21)	2017	Bilateral RDN plus baseline AHM	Baseline AHM	Symplicity Flex	MRA	≤2 mins	≤8 W	4–6
Peters et al. (11)	2017	Bilateral RDN plus baseline AHM	Sham procedure plus baseline AHM	Symplicity Flex	MRA	2 mins	5–8 W	4–6
Rosa et al. (22)	2017	Bilateral RDN plus baseline AHM	Baseline AHM plus spironolactone	Symplicity Flex	MRA	NA	8 W	4–6
Celinska et al. (23)	2018	Bilateral RDN plus baseline AHM	Baseline AHM	Symplicity Flex	MRA	≤2 mins	≤8 W	≤6
Engholm et al. (24)	2018	Bilateral RDN plus baseline AHM	Sham procedure plus baseline AHM	Symplicity Flex	MRA	2 mins	5–8 W	4–6
Schmieder et al. (25)	2018	Bilateral RDN plus baseline AHM	Sham procedure plus baseline AHM	Kona Surround Sound	MRA	3 min	NA	14
Sexena et al. (26)	2018	Bilateral RDN plus baseline AHM	Sham procedure plus baseline AHM	Kona Surround Sound	MRA	3 min	NA	14
Oliveras et al. (27)	2018	Bilateral RDN plus baseline AHM	Baseline AHM plus spironolactone	Symplicity Flex	MRA	NA	≤8 W	4–6
Böhm et al. (10)	2019	Bilateral RDN only	Sham procedure only	Symplicity Spyral	MRA plus SB	NA	NA	NA
Lurz et al. (28)	2020	Bilateral RDN plus baseline AHM	Sham procedure plus baseline AHM	Symplicity Flex/ Spyral/Paradise	MRA	2 mins	NA	4–6
Ukena et al. (20)	2020	Bilateral RDN plus baseline AHM	NA	Symplicity Flex	MRA	≤2 mins	≤8 W	4–6

*AHM, antihypertensive medications; MRA, main renal artery; SB, side branch; NA, not available*.

The 16 studies including 681 patients reported different HR types involving office HR, 24 h-HR, daytime HR, and nighttime HR. The mean HR of baseline was between 60 and 80 bpm, and the SBP at baseline was higher than 140 mmHg except the study by Fengler et al. (19). The extracted results are shown in Table 3.

**Table 3:** Extracted data of the included studies.

**References**	**Year**	**Heart rate (bpm)**			**Systolic blood pressure (mmHg)**		
		**Baseline**	**Change**	***P* value**	**Baseline**	**Change**	***P* value**
**Office measurement**							
Mahfound et al. (16)	2011	69.7 ± 2.0	−3.4 ± 1.5	0.082	177 ± 3	−32 ± 4	<0.001
Ukena et al. (17)	2011	73 ± 14	−4 ± 11	0.028	172 ± 24	−31 ± 19	<0.0001
Ukena et al. (18)	2013	66.1 ± 1.0	−2.6 ± 1.0	0.001	176.7 ± 1.8	– 25.5 ± 2.4	<0.0001
Fengler et al. (19)	2016	67.4 ± 10.9	2.2 ± 7.5	0.09	132.8 ± 16.1	−3.0 ± 19.1	0.24
Rosa et al. (22)	2017	71 ± 14	−4.5 ± 12.7	0.49	159 ± 19	−17.7 ± 22.3	0.001
Sexena et al. (26)	2018	78.5 ± 13.0	−6.0 ± 11.5	0.03	170.7 ± 11.2	−16.1 ± 27.3	0.04
Schmieder et al. (25)	2018	68.4 ± 12.1	0.2 ± 8.4	NA	181.1 ± 19.7	−12.8 ± 26.0	NA
Celinska et al. (23)	2018	72 ± 11	−2.0 ± 10.7	0.36	164 ± 16	−22 ± 24	<0.001
Oliveras et al. (27)	2018	67.1 ± 10.6	0.9 ± 23.5	NA	168.0 ± 13.8	−17.5 ± 18.3	NA
Lurz et al. (28)	2020	59.1 ± 11.1	4.7 ± 8.3	NA	144.8 ± 4.8	−8.8 ± 8.0	NA
Other measurements^*^							
Kiuchi et al. (20)	2016	76 ± 16	−3 ± 6	NA	NA	NA	NA
Courand et al. (21)	2017	74.1 ± 11.0	−6.7 ± 7.3	NA	159.0 ± 22.1	−23.7 ± 17.5	NA
Peters et al. (11)	2017	NA	0 ± 11.1	0.54	151 ± 13	−5 ± 17	0.18
Engholm et al. (24)	2018	70.6 ± 2.1	−0.3 ± 1.4	NA	151.5 ± 2.2	−5.0 ± 3.0	NA
Böhm et al. (10)	2019	72.9 ± 11.0	−2.5 ± 5.3	NA	153.5 ± 9.2	−5.5 ± 10.3	NA
Ukena et al. (29)	2020	65.7 ± 9.9	−0.4 ± 6.7	0.772	148.3 ± 20.4	−7.8 ± 18.6	<0.001

*NA, not available; other measurements*

* *:24-h heart rate (HR), daytime HR, nighttime HR*.

### Included Studies Quality Evaluation

**Figure 2:**
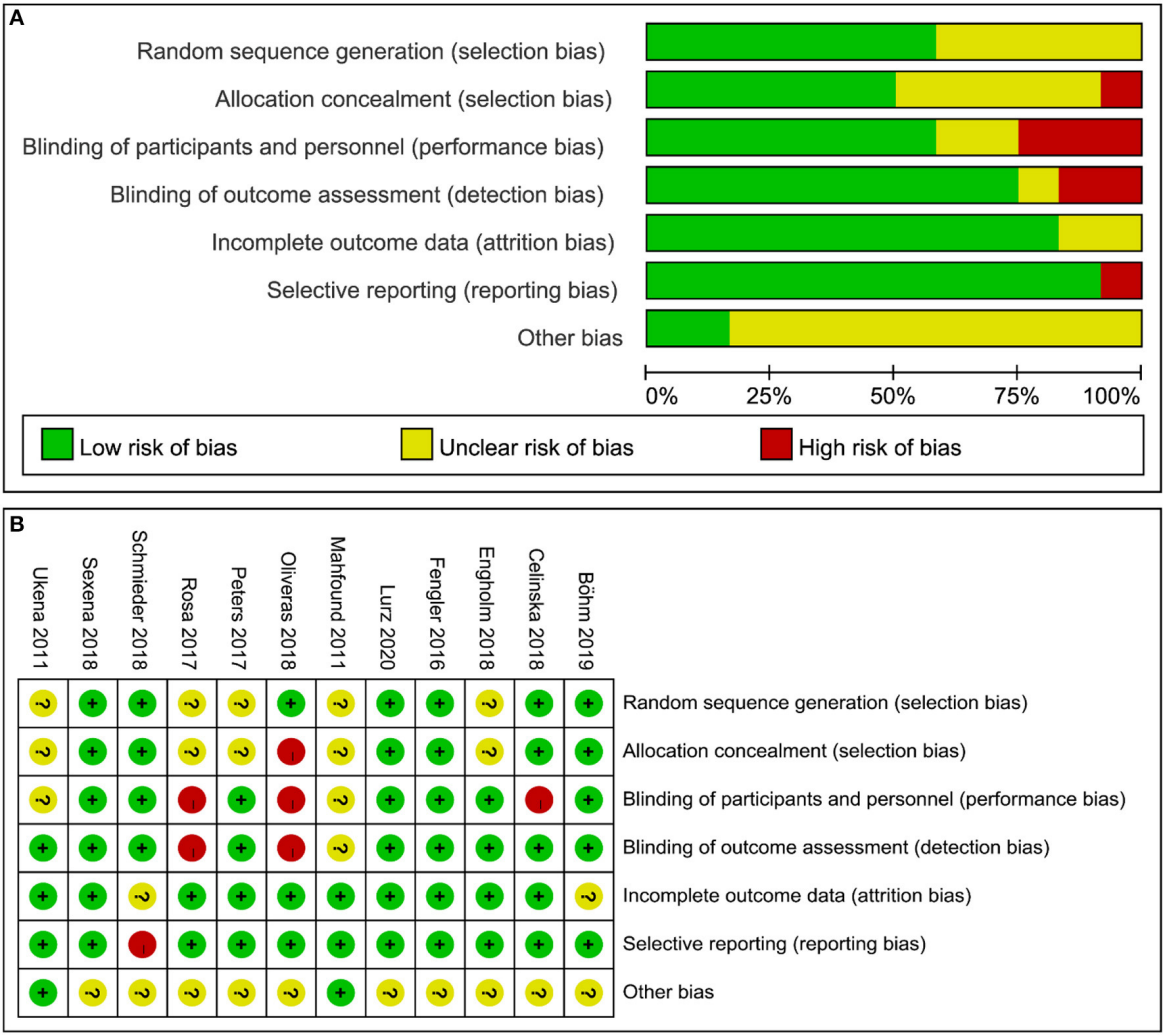
Quality evaluation of the included studies. **(A)** Review authors' judgments presented as percentages for the included studies; **(B)** Review authors' judgements for each included study.

The quality evaluation of thirteen RCTs was assessed by Cochrane Collaboration's tool, the results are shown in Figures 2A,B. Three non-RCTs were included in this meta-analysis, methodological index for non-randomized studies (MINORSs) tool with a total score of 16 was used to evaluate the literature quality with a score of 14 for the study by Ukena et al. (18), 13 for the study by Kiuchi et al. (20), and 14 for the study by Ukena et al. (29).

### Results of Outcomes

#### Primary Outcomes

**Figure 3:**
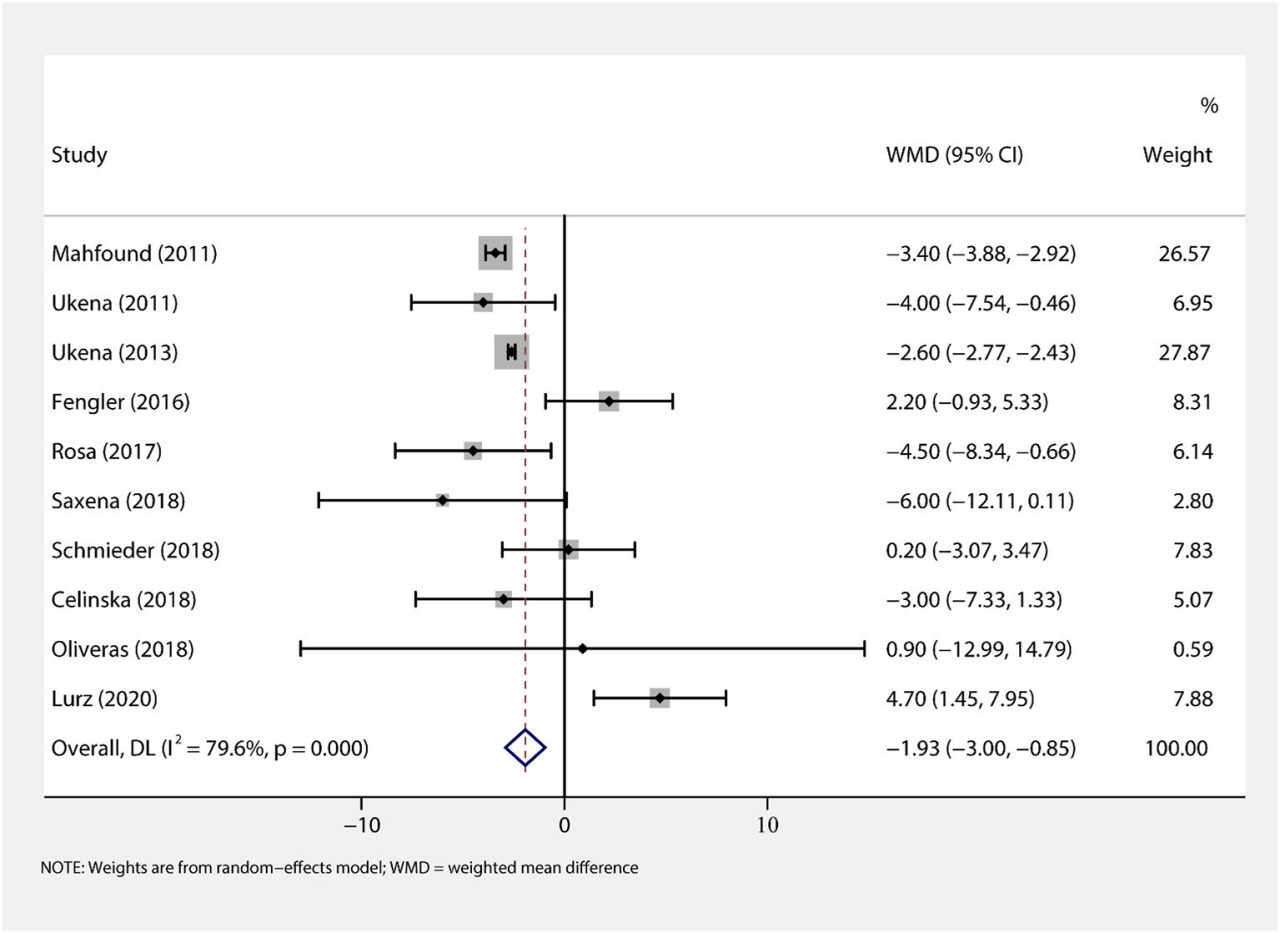
The forest plot of office heart rate (HR) change from baseline. Weights are from random-effects model. WMD, weighted mean difference.

We performed a meta-analysis of data from 10 studies that included 395 participants with complete office HR data (Figure 3), mean follow-up was 6.3 months, and found that RDN caused a significant drop of office HR compared with baseline (WMD = −1.93, 95% *CI*: −3.00 to −0.85, *p* < 0.001). As the heterogeneity is high between the studies (*I*^2^ = 79.6%, *p* < 0.001), we used the random-effects model to analyze the effect.

Besides, we conducted subgroup analysis to identify the effect of covariables on the primary outcome.

**Figure 4:**
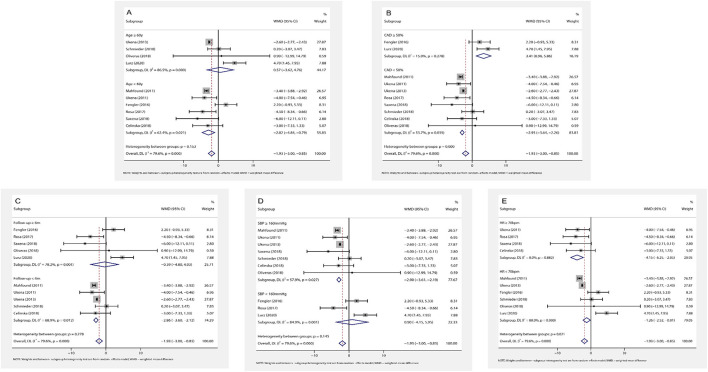
The forest plot of subgroup analysis: **(A)** by age; **(B)** by the prevalence of coronary artery disease (CAD); **(C)** by the length of follow-up; **(D)** by baseline systolic blood pressure (SBP); **(E)** by baseline HR. Weights and between subgroup heterogeneity test are from random-effects model. WMD, weighted mean difference.

(1) Association of age and HR change: subgroup analysis results by age (age ≥ 60 or < 60 years) are shown in Figure 4A. RDN in the population aged < 60 years caused a statistically reduction of office HR (WMD = −2.82, 95% *CI*: −4.84 to −0.79, *p* = 0.006). Additionally, RDN may not affect the office HR in the elders (WMD = 0.57, 95% *CI*: −3.62 to 4.76, *p* = 0.789).

(2) Association of CAD and HR change: in the patients with frequent CAD history (≥ 50% participants), RDN slightly increased office HR (WMD = 3.41, 95% *CI*: 0.96–5.86, *p* = 0.006). On the contrary, RDN in the population with infrequent CAD history (< 50% participants) shown a significant reduction of office HR (WMD = −2.95, 95% *CI*: −3.64 to −2.26, *p* < 0.001) (Figure 4B).

(3) Association of follow-up time and HR change: the included trials were divided into two groups by the length of follow-up (≥ 6 or < 6 months). RDN caused a statistically reduction of office HR in the short follow-up group (WMD = −2.86, 95% *CI*: −3.60 to −2.12, *p* < 0.001). On the other hand, the office HR in the long follow-up group may not be influenced by RDN (WMD = −0.39, 95% *CI*: −4.80 to 4.03, *p* = 0.864) (Figure 4C).

(4) Association of baseline SBP and HR change: according to the current classification of hypertension (30) and baseline SBP, we divided the trials into grade one hypertension (SBP: 140–159 mmHg) and grade 2–3 hypertension (SBP ≥ 160 mmHg) group. We found that the grade one hypertension group had no significant change of office HR (WMD = 0.90, 95% *CI*: −4.15 to 5.95, *p* = 0.727), but RDN caused the statistically drop in another group (WMD = −2.90, 95% *CI*: −3.61 to −2.19, *p* < 0.001) (Figure 4D).

(5) Association of baseline HR and HR change: in the high baseline HR group (baseline HR ≥ 70 bpm), RDN significantly reduce the office HR (WMD = −4.15, 95% *CI*: −6.25 to −2.05, *p* < 0.001). This influence shown in the low baseline HR group (baseline HR < 70 bpm) with WMD = −1.26 (95% *CI*: −2.51 to −0.01, *p* = 0.048) (Figure 4E).

#### Secondary Outcomes

First, in the nine studies (*n* = 379) with complete 24 h-HR testing data, RDN showed a significant reduction of 24-h HR (WMD = −1.73, 95% *CI*: −3.51 to −0.31, *p* = 0.017) (Supplementary Figure 1). Second, we collected the daytime HR data from six studies with 199 participants and found that RDN could decrease the daytime HR as well (WMD = −2.67, 95% *CI*: −5.02 to −0.32, *p* = 0.026) (Supplementary Figure 2). Third, we analyzed the nighttime HR lowering effect of RDN in six related trials (*n* = 262) and found that RDN did not significantly affect the nighttime HR (WMD = −2.08, 95% *CI*: −4.57 to 0.42, *p* = 0.103) (Supplementary Figure 3). Finally, in the ten studies that included OSBP records (*n* = 395), we conducted a meta-analysis to identify the correlation of the HR reduction with the SBP decrease, on the one hand, RDN could significantly reduce the OSBP (WMD = −25.56, 95% *CI*: −29.34 to −21.78, *p* < 0.001) (Supplementary Figure 4), on the other hand, we conducted a Spearman's correlation analysis and found that the reduction of HR was highly related to the decrease of SBP (*r* = 0.658, *p* < 0.05).

### Heterogeneity Analysis

**Figure 5:**
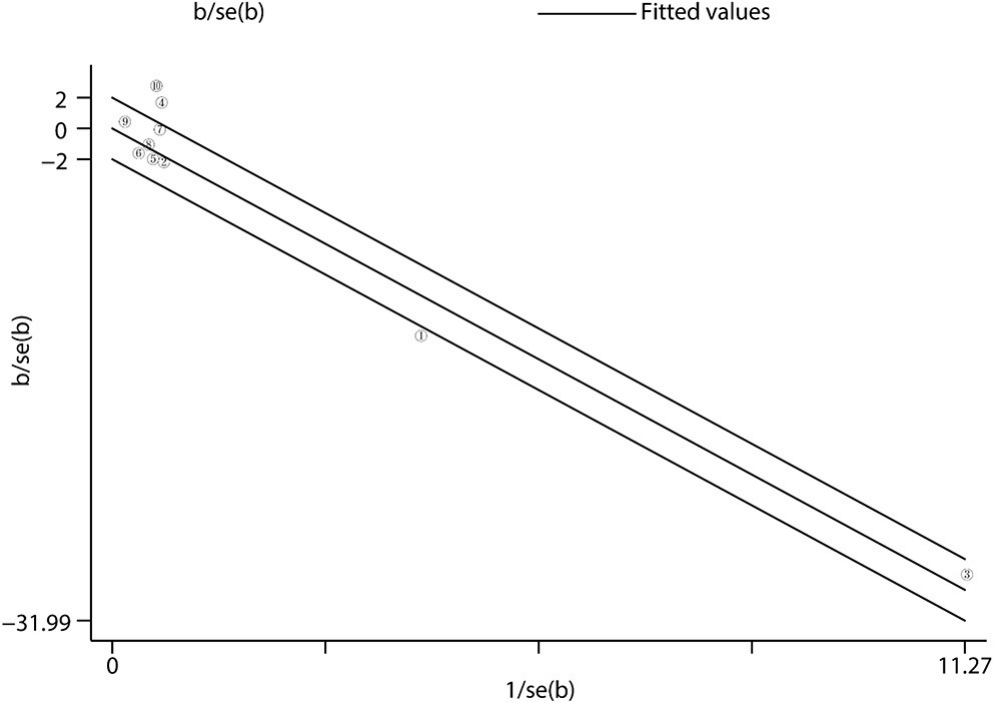
Heterogeneity analysis. Heterogeneity was evaluated by Galbraith radial plot. The office measurement studies were included for the analysis. = Mahfound et al. (16) … = Lurz et al. (28).

There was a high heterogeneity for the primary outcome analysis (*I*^2^ = 79.6%, *p* < 0.001). Therefore, we conducted a meta-regression analysis of variables, such as age, prevalence of CAD, follow-up time, baseline HR, and SBP to identify the main sources of heterogeneity. The results suggested that the prevalence of CAD was the main factor of heterogeneity with a regression coefficient of −6.89 (95% *CI*: −13.40 to −0.37, *p* = 0.043), and other variables could not explain the source of heterogeneity. In the subgroup analysis, we found that when the prevalence of CAD was considered, the heterogeneity was reduced with *I*^2^ = 15.0% in the frequent CAD group (*p* = 0.278) and *I*^2^ = 53.7% in the infrequent group (*p* = 0.035) (Figure 3B). In addition, in the baseline HR subgroup, the heterogeneity was reduced with *I*^2^ = 0% (*p* = 0.882) when baseline HR ≥ 70 bpm. However, the heterogeneity did not significantly decrease in other subgroups. Furthermore, we performed the Galbraith radial plot to identify the inter-study heterogeneity and found that three studies by Mahfound et al. (16), Fengler et al. (19), and Lurz et al. (28) may be the sources of the heterogeneity (Figure 5).

### Sensitivity Analysis

**Figure 6:**
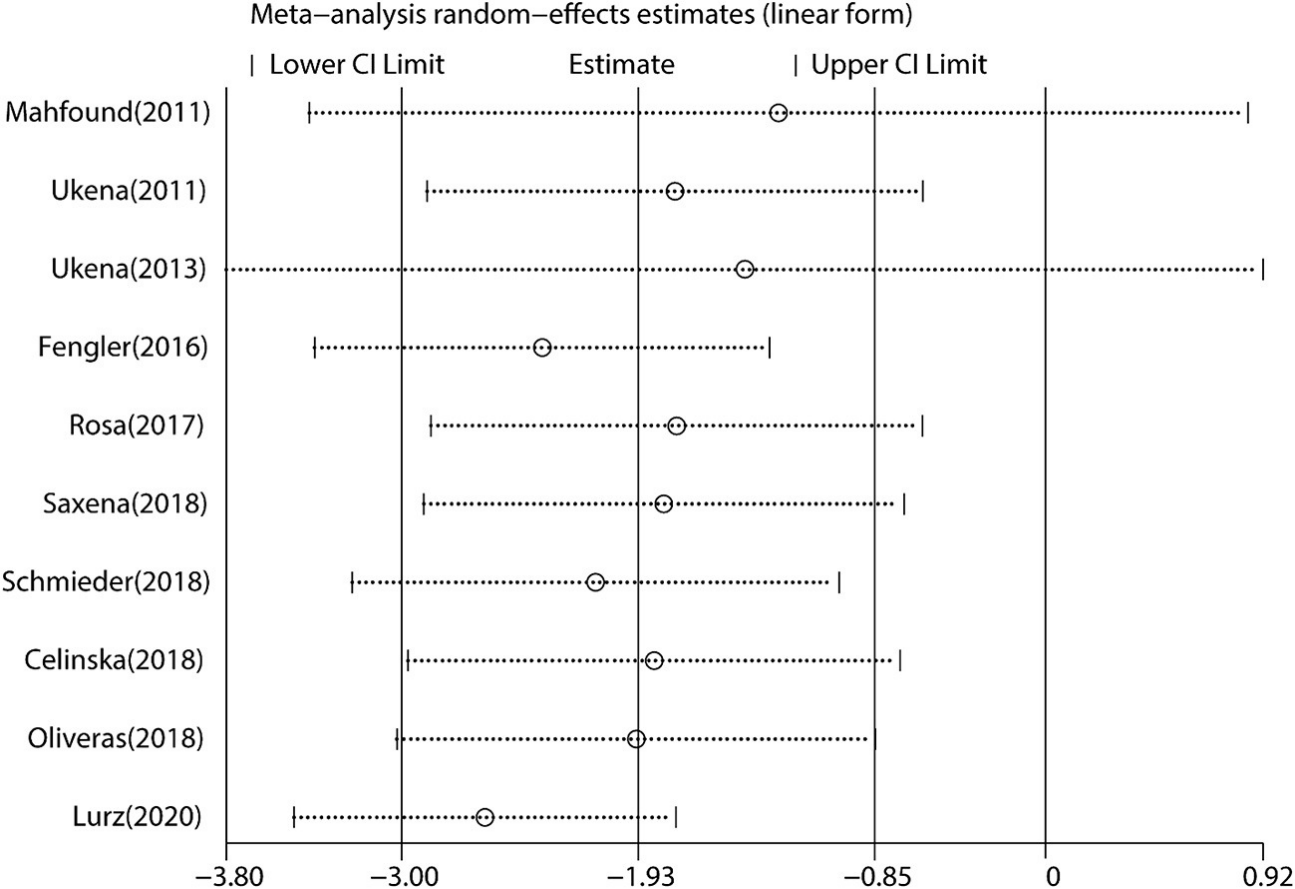
Sensitivity analysis.

The sensitivity analysis of the data from the primary outcome analysis showed that the exclusion of studies by Mahfound et al. (16) and Ukena et al. (18) highly affected the main results of the meta-analysis (Figure 6).

### Publication Bias

**Figure 7:**
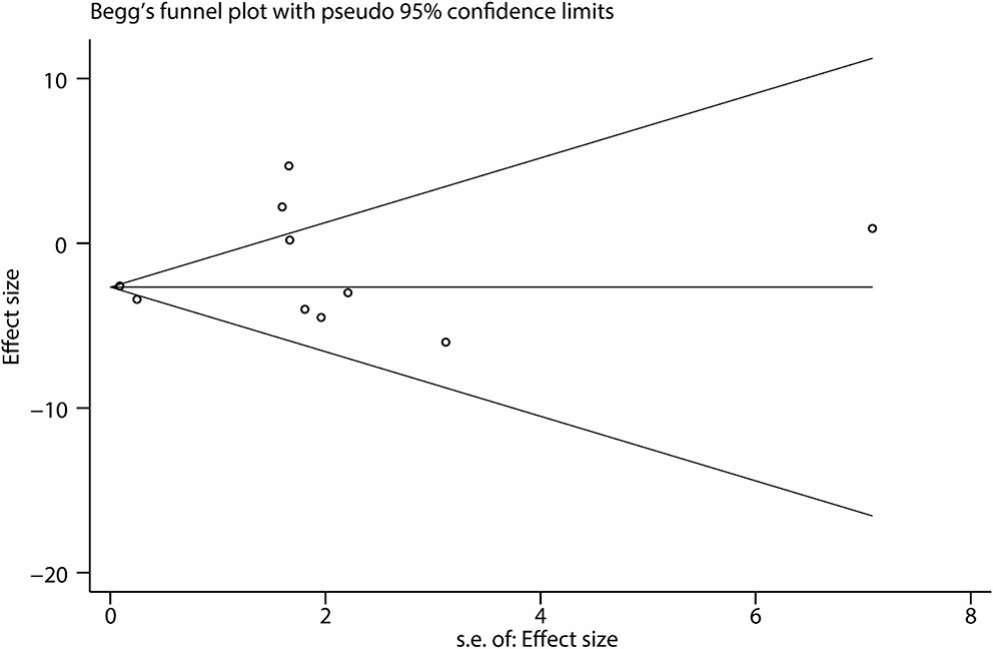
Publish bias analysis. Publish bias was evaluated by Begg's funnel plot.

Begg's funnel plot showed that there was little publication bias in the included studies (Figure 7). We could not find any evidence of publication bias by Egger's test (bias coefficient = 0.60, 95% *CI*: −1.32 to 2.52, *p* = 0.492).

## Discussion

Although the precise mechanisms of RDN are not fully understood, it is believed that RDN may exert hypotension, anti-arrhythmia, and attenuating heart failure effects by inhibiting sympathetic overactivation (31), and thereby reducing plasma concentrations of norepinephrine (32), renin, and angiotensin (33). Basal HR may reflect systemic sympathetic activity to some extent. However, controversy still exists regarding the role of RDN in HR control. To our knowledge, this study is the first meta-analysis to study the effect of RDN on HR, and based on the results we report the main findings as followings: (1) RDN can effectively reduce HR; (2) RDN may have a stronger HR control effect in patients with low age, no history of CAD, high baseline BP, and HR; (3) RDN mainly controls daytime HR and may not have an effect on nighttime HR; and (4) the HR lowering effect of RDN is closely related to the decrease of BP, suggesting that the mechanisms of action of both may be the same.

Heart rate is a key predictor of adverse events in a variety of cardiovascular and non-cardiovascular diseases (34), and high baseline HR is strongly associated with events, such as heart failure and stroke. On the contrary, cardiovascular benefits are associated with HR control (35). Although Engholm et al. (24) concluded that RDN did not have a significant effect on HR, but 83% of the participants were treated with beta-blocker, which may influence the effectiveness of RDN on HR control. To exclude drug interference, Böhm et al. (10) demonstrated in patients without receiving antihypertensive drugs that RDN reduced HR independently of drugs, with a −2.7 bpm (95% *CI*: −4.5 to −1.0, *p* = 0.003) change in 24-h HR in the RDN group compared with the sham-controlled group. In the present study, although most of the included participants were treated with beta-blocker (Table 2), RDN significantly reduced HR compared with the baseline, further confirming the HR lowering effect of RDN. In addition, although recently a meta-analysis with limited studies demonstrated that RDN may induce bradycardia [relative risk (*RR*) = 6.63, 95% *CI*: 1.19–36.84, *p* = 0.03] (36). In the present meta-analysis, only one study reported the adverse effect with 5 (5/30) patients who suffered from bradycardia after RDN (23), it may be related to the significant and fast decreased sympathetic activity, and whether bradycardia was a side effect of RDN should be demonstrated by large-scale RCTs.

Isolated systolic hypertension (ISH) is the most common type of hypertension in elderly patients, and is characterized as reduced elastic arterial compliance and vascular endothelial dysfunction; sympathetic hyper-activation has limited influence on ISH, (37), therefore, RDN may have a limited effect in patients with ISH. Fengler et al. (38) concluded that compared to patients with ISH, patients with non-ISH were better suited to receive RDN, and after 3 months of follow-up, the reductions of office SBP were −5.9 ± 11.8 and −13.3 ± 11.7 mmHg (*p* = 0.001), respectively. In addition, the present study found that RDN was effective in reducing HR in patients with hypertension aged <60 years (WMD = −2.82, *p* < 0.001), but had no effect on HR in patients aged ≥60 years (WMD = 0.57, *p* = 0.789). However, elderly patients are more likely to be suffered from comorbidities, such as diabetes mellitus and heart failure, and elders often receive multiple drugs, these factors may have an influence on the effectiveness of RDN. Due to the lack of enough data, we did not adjust the results with the factors, therefore, the results should be interpreted with caution. In addition, this study found that RDN was more effective in controlling HR in studies with a lower prevalence of CAD. Although the relationship between sympathetic activation and CAD remains incompletely understood, but based on the results of this study, we suggest that RDN may be more effective in controlling HR in non-CAD patients.

Increased baseline BP and HR are associated with the increased sympathetic activity (33), and can predict BP reduction after RDN (31). Böhm et al. (10) suggested that there was a significant difference of BP change between RDN and sham group at HRs ≥ 73.5 bpm, but not for patients with HRs < 73.5 bpm. In the present study, the reduction of HR by RDN was more significant in patients with baseline BP ≥ 160 mmHg compared to those with baseline BP < 160 mmHg, with a WMD of −2.90 (95% *CI*: −3.61 to −2.19, *p* < 0.001) vs. 0.90 (95% *CI*: −4.15 to 5.95, *p* = 0.727). Similarly, the HR-lowering effect of RDN was stronger for patients with baseline HR ≥ 70 bpm. In addition, we verified the correlation between SBP reduction and decreased HR and showed that the reduction of HR was strongly associated with the drop of SBP. Although whether RDN can reduce HR by suppressing sympathetic overactivation was unclear, but we found that RDN could reduce both HR and BP simultaneously.

The long-term effectiveness of the hypotensive and other effects of RDN are not fully validated in RCTs, and the mostly follow-up period of current clinical studies on RDN is within 6 months. Although the 3-year follow-up results of the Global Symplicity Registry (GSR) study showed that the hypotensive effect of RDN were persistent (39), but previous animal trials have found that there was chronic anatomical and functional reinnervation of renal sympathetic nerve fibers after RDN (40). Further research is needed to determine the long-term value. In the present study, we found that the effect of RDN on HR was also affected by the duration of follow-up. There was a more significant decrease in HR in the subgroup with follow-up <6 months than the subgroup with follow-up ≥6 months. This observation needs to be supported by long-term follow-up evidence from RCTs.

In addition, we analyzed the effects of RDN on 24-h HR, daytime HR, and nighttime HR and found that RDN significantly decreased 24-h HR, daytime HR, but nighttime HR did not seem to be affected by RDN, which was consistent with the findings of Böhm et al. (10). The circadian rhythm of sympathetic nerves in normal subjects is higher at the day than at night (41), and inappropriate elevations in nocturnal sympathetic activity can cause nocturnal hypertension and increase HR, which are significantly associated with a high risk of cardiovascular events (42). The dominance of vagus rather than sympathetic nerves at night may contribute to the poor control of nocturnal HR by RDN.

Although our study found some contribution of RDN to HR control, its precise mechanism of action is still unclear, and it remains uncertain whether this effect is related to the decrease in sympathetic activity induced by RDN. In addition, the optimal population for RDN, the long-term efficacy of RDN for HR control, and whether cardiovascular benefits can be derived from RDN for HR control remain unclear, and future clinical studies should aim to address the above questions.

## Limitations

Several limitations should be considered. First, although 16 studies were included in this meta-analysis, the sample sizes of the studies were small which may have effect on the results. Second, there were important data that could not be obtained, such as the use of beta-blocker, and a subgroup analysis of which would be important for the analysis of the study findings. Finally, although we included studies in the literature based on strict inclusion and exclusion criteria, some of the included literature still had limitations in the study design, and there was a certain risk of bias. For example, the implementation of allocation concealment was not mentioned in some included studies, which increased the possibility of human factor intervention and caused selection bias. In addition, there was intra- and inter-study heterogeneity in the included literature. Therefore, the results of this study should be interpreted with caution, and more clinical trials are needed in the future to draw definitive conclusions.

## Conclusion

In conclusion, RDN could reduce HR except at night and is highly associated with BP control. These findings suggest that sympathetic regulation by RDN involves HR reduction which may have cardiovascular benefits. Future clinical trials with large numbers of patients are needed to demonstrate this conclusion.

## Data Availability Statement

The original contributions presented in the study are included in the article/Supplementary Material, further inquiries can be directed to the corresponding author.

## Author Contributions

This study was designed by LL. ZH and YLX were responsible for data collection and statistical analysis. LL wrote the first draft. YY reviewed and checked the manuscript. All authors read and approved the final manuscript.

## Funding

This study was supported by the National Natural Science Foundation of China (Grant Number: 81970285).

## Conflict of Interest

The authors declare that the research was conducted in the absence of any commercial or financial relationships that could be construed as a potential conflict of interest.

## Publisher's Note

All claims expressed in this article are solely those of the authors and do not necessarily represent those of their affiliated organizations, or those of the publisher, the editors and the reviewers. Any product that may be evaluated in this article, or claim that may be made by its manufacturer, is not guaranteed or endorsed by the publisher.
