# Supplementary material for: Effect of Renal Denervation for the Management of Heart Rate in Patients With Hypertension: A Systematic Review and Meta-Analysis
Source: Front Cardiovasc Med. 2022 Jan 17;8:810321. doi: 10.3389/fcvm.2021.810321 (PMC8801499; doi:10.3389/fcvm.2021.810321)
Supplement: Supplementary file 1 [file Data_Sheet_1.docx]

**Effect of renal denervation for the management of heart rate in Patients with hypertension: A systematic review and meta-analysis.**

Supplementary Material

# Supplementary Figures


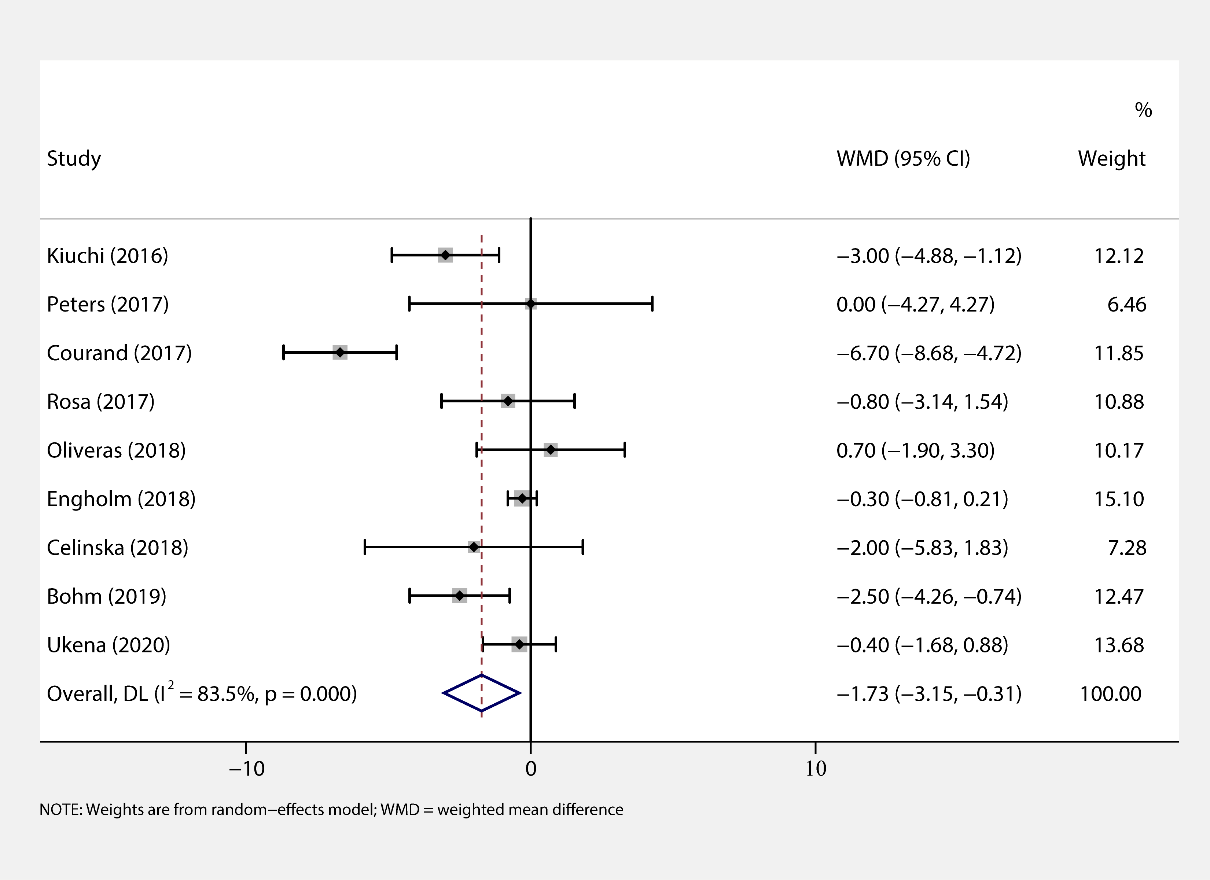


**Supplementary Figure 1.** The forest plot of 24-hour heart rate change from baseline.


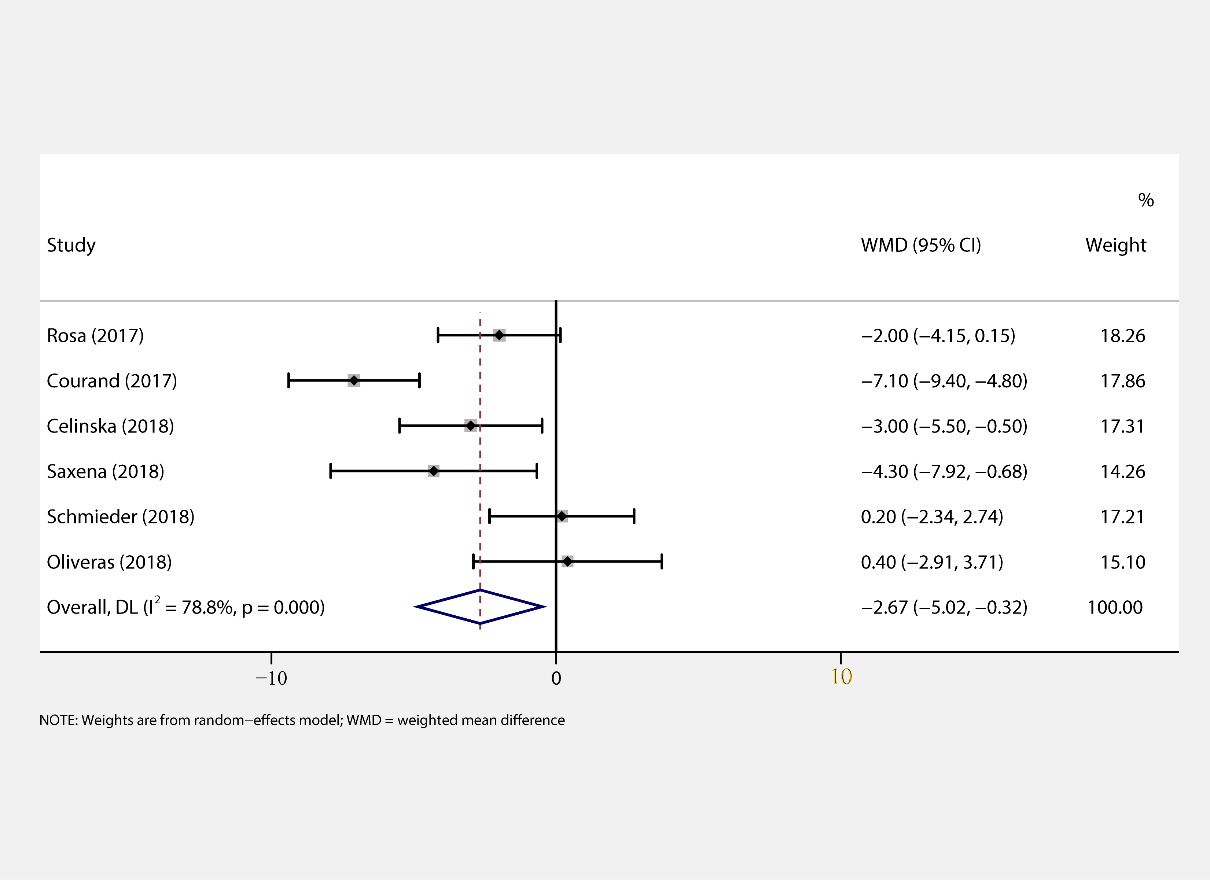


**Supplementary Figure 2.** The forest plot of daytime heart rate change from baseline.


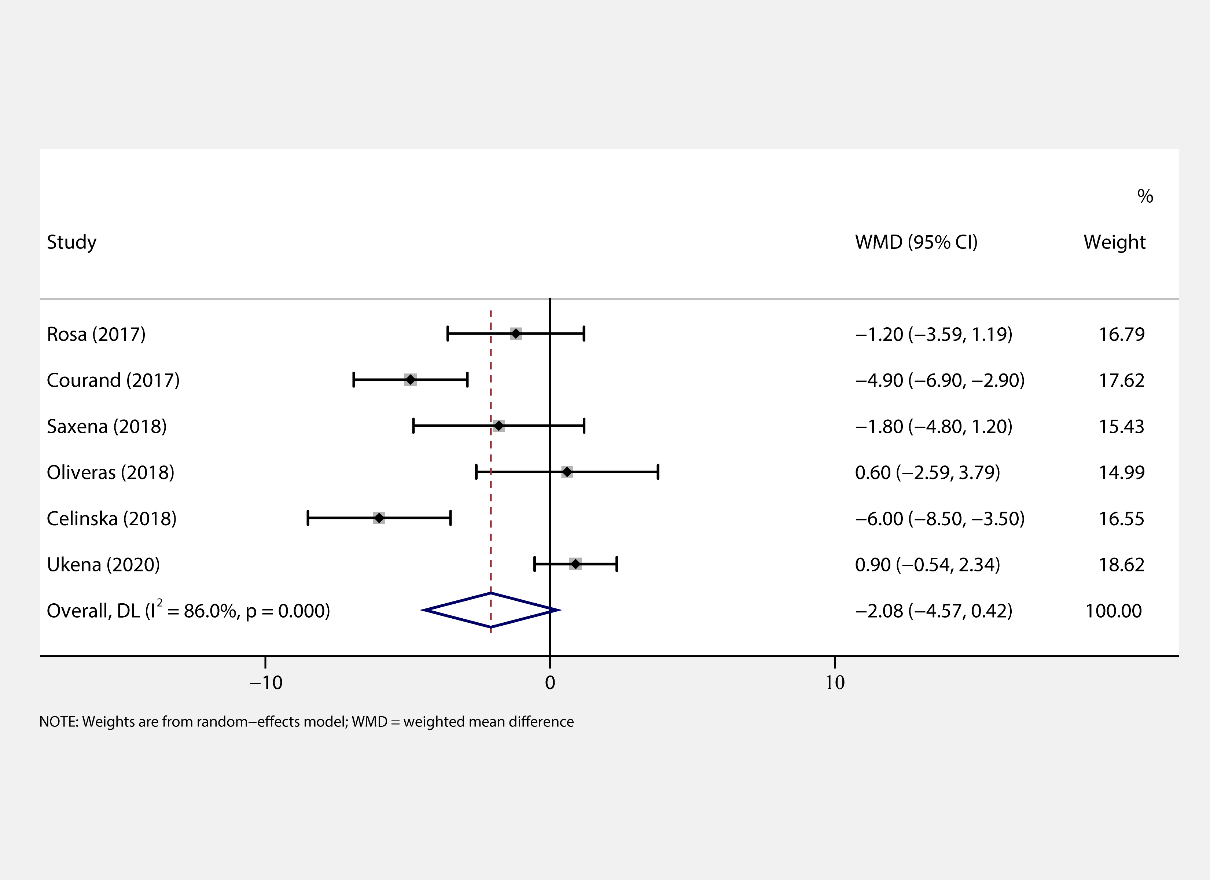


**Supplementary Figure 3.** The forest plot of nighttime heart rate change from baseline.


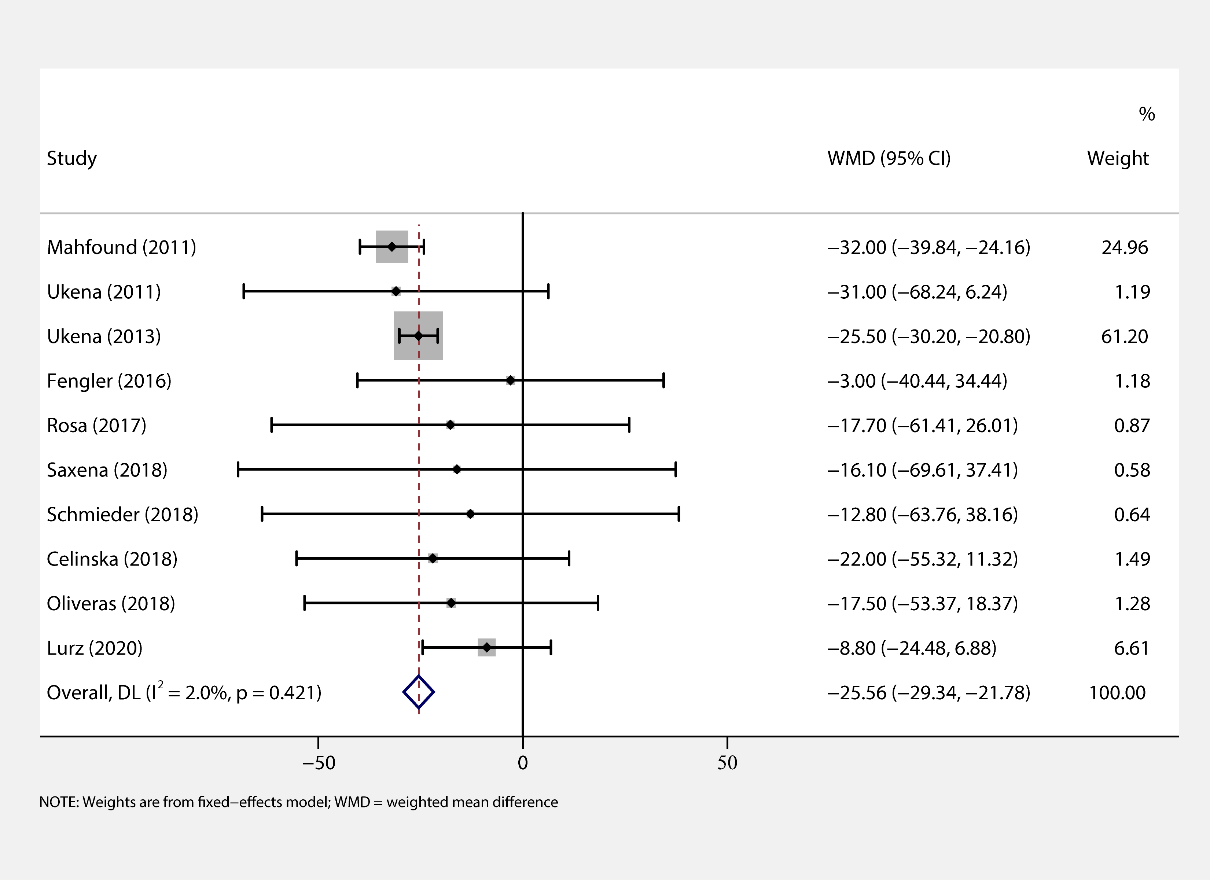


**Supplementary Figure 4.** The forest plot of office systolic blood pressure change from baseline.
